# Controlled and Local Delivery of Antibiotics by 3D Core/Shell Printed Hydrogel Scaffolds to Treat Soft Tissue Infections

**DOI:** 10.3390/pharmaceutics13122151

**Published:** 2021-12-14

**Authors:** Ashwini Rahul Akkineni, Janina Spangenberg, Michael Geissler, Saskia Reichelt, Hubert Buechner, Anja Lode, Michael Gelinsky

**Affiliations:** 1 Centre for Translational Bone, Joint and Soft Tissue Research, University Hospital Carl Gustav Carus, Faculty of Medicine of Technische Universität Dresden, 01307 Dresden, Germany; JSpangenberg90@aol.com (J.S.); michael.geissler@tu-dresden.de (M.G.); anja.lode@tu-dresden.de (A.L.); michael.gelinsky@tu-dresden.de (M.G.); 2 Institute of Natural Materials Technology, Technische Universität Dresden, 01069 Dresden, Germany; saskia.reichelt@tu-dresden.de; 3 Heraeus Medical GmbH, 61273 Wehrheim, Germany; hubert.buechner@heraeus.com

**Keywords:** 3D core/shell printing, drug delivery, antibiotics, hydrogels, soft tissue infection

## Abstract

Soft tissue infections in open fractures or burns are major cause for high morbidity in trauma patients. Sustained, long-term and localized delivery of antimicrobial agents is needed for early eradication of these infections. Traditional (topical or systemic) antibiotic delivery methods are associated with a variety of problems, including their long-term unavailability and possible low local concentration. Novel approaches for antibiotic delivery via wound coverage/healing scaffolds are constantly being developed. Many of these approaches are associated with burst release and thus seldom maintain long-term inhibitory concentrations. Using 3D core/shell extrusion printing, scaffolds consisting of antibiotic depot (in the core composed of low concentrated biomaterial ink 3% alginate) surrounded by a denser biomaterial ink (shell) were fabricated. Denser biomaterial ink (composed of alginate and methylcellulose or alginate, methylcellulose and Laponite) retained scaffold shape and modulated antibiotic release kinetics. Release of antibiotics was observed over seven days, indicating sustained release characteristics and maintenance of potency. Inclusion of Laponite in shell, significantly reduced burst release of antibiotics. Additionally, the effect of shell thickness on release kinetics was demonstrated. Amalgamation of such a modular delivery system with other biofabrication methods could potentially open new strategies to simultaneously treat soft tissue infections and aid wound regeneration.

## 1. Introduction

Patients with open fractures and severe burns (with >20% burned body surface), are often presented in clinics with extensive soft tissue damage. Such soft tissue wounds are often at a high risk of infection, as the soft tissue of the skin is directly exposed to contaminants after a high-energy trauma (e.g., road or fire accidents). These contaminants and/or surgical treatment procedures can introduce microorganisms at the damaged soft tissue, potentially leading to an invasive infection [1]. In case of soft tissue damage by chronic burn wounds, therapeutic interventions that target management of inflammation, pain and prevent or treat infections caused by the invasive colonization of resident microorganisms due to immune dysregulation are essential [2]. Additionally, for open fractures, prevention of infections in the damaged soft tissue from the contaminants and resident microorganisms are deemed essential for successful fracture healing and restoration of function [1]. Furthermore, reconstructive surgical intervention might be needed depending on the severity of the wound to aid the healing process [3,4].

Infections that develop in soft tissues after an extensive trauma or burn injury are the predominant reason for complications, morbidity, and mortality [1,2,5,6,7]. Critical bacterial concentrations (>10^5^ colony forming units) [2] at the burn wound site, most frequently of Staphylococcus aureus (*S. aureus*, Gram-positive, opportunistic bacteria), Pseudomonas aeruginosa and Acinetobacter (Gram-negative), are reported to be the most common cause of death in burn patients [2]. Additionally, primary infection of soft tissue in open fractures is majorly associated with *S. aureus* [8], potentially leading to osteomyelitis and other morbidities (1–42.9% of all patients) [1,9]. Bacterial infections in damaged soft tissues lead to a plethora of detrimental events such as systemic infection in the patient, sepsis, graft loss, delayed healing, higher treatment costs and increased mortality [1,2]. Hence, the primary concern in the management of open fractures and burn wounds is prevention of infection [1,9,10].

Currently, clinical care of infected soft tissues in open fractures and burns includes systemic and local treatments for managing infection at the injury site along with facilitating regeneration of bone [4,9] and skin regeneration (by excision and grafting with allografts or skin substitutes) [10,11]. When patients with open fractures or burn wounds are presented in the hospital, many antibiotics (penicillin derivatives, cephalosporins, aminoglycosides, etc.) can be systemically administered along with irrigation and debridement of the trauma site to avoid/reduce development of infections [4,12]. Traditional, systemic administration of antibiotics is usually associated with inadequate availability of the drug locally and thus may not be effective to deter aggressive infections and can also lead to many side effects, such as drug induced toxicity in other tissues. Inhibitory concentration of antibiotics can be achieved by local application, leading to effective management of the infection at the injury site [13,14,15,16]. Local delivery of antibiotics in open fracture patients is usually achieved by antibiotic-loaded wound coverages such as collagen fleeces [17], antibiotic-loaded PMMA (poly (methyl methacrylate)) bead chains or antibiotic coated metal implants [9]. In case of burn wounds, topical application of antibiotics lotions at the injury site and antibiotic-loaded wound coverages (composed of various hydrogels) are routinely used [18,19]. Locally delivered antibiotics will develop effective concentration only at the infected site, thus directing their action specifically only at the infected site, therefore avoiding systemic toxicity. Additionally, studies have shown that a very high concentration of antibiotics (that are toxic when delivered systemically) can be released locally by hydrogels without adverse effects at the site of infection [18].

However, as the concentration of the locally applied antibiotics drastically reduces in a short time, falling below minimum inhibitory concentration (MIC), multiple applications of the antibiotic at the injury site are usually required to completely eradicate the infection and its recurrence. As a result of such a procedure, local antibiotic concentrations vary over a wide range, leading to low efficiency in treating the infection. Additionally, as the local application of antibiotics involves physical contact with the wound, multiple applications can potentially interfere with healing of the wound and can introduce new infection, in addition to the excruciating pain experienced by the patient during such a treatment [13].

Searching for alternatives, the development of new approaches to sustainably deliver antibiotics at soft tissue infection sites—hydrogels, nanofibers, and nanoparticles, etc.—are being actively pursued [20]. Many synthetic and naturally occurring hydrogels loaded with antibiotics have been studied for their applicability in treating soft tissue infections [21]. Hydrogels, applied as wound dressings, can be usually loaded with the desired amount of antibiotics and, to an extent, their release can be controlled. As hydrogels are mostly composed of aqueous solutions, they help in maintaining a moist environment—thus aiding in wound healing by promoting cell recruitment, adhesion, proliferation, cell-growth factor interactions and angiogenesis [18]. Furthermore, fabrication of hydrogels and loading of antibiotics is a rather simple process. Though hydrogels can release antibiotics over an extended period of time, lack of mechanical strength deters their extensive use in treating chronic burn wounds, especially in treatments involving reconstructive surgeries. The application of nanofibrous mats fabricated by electrospinning methods that can both load and deliver antibiotics as wound dressings has also been studied. The very high surface to volume ratio of these systems provides the unique capability to sustainably deliver antibiotics over extended time periods. Furthermore, they could provide mechanical stability and their functionality can be enhanced by various surface modification procedures. However, toxic solvents usually used for electrospinning and complications arising from large scale production could be probable reasons for deterring clinical use [11]. Nanoparticle carrier systems for drug delivery have also been extensively studied to treat infections. Nanoparticle systems could potentially extend drug half-life, thus improving pharmacokinetics [20]. Lipid based and polymeric nanoparticle systems have been used to encapsulate various antibiotics. Lipid based and cationic polymer nanoparticles have high mucoadhesive properties at the wound site, allowing more time for the encapsulated drug to release. The ease of application, high bioavailability and their specificity are highly advantageous in treating soft tissue infections. Though nanoparticle carrier systems have many intrinsic advantages, their application can be envisioned only for superficial infections due to particle coalescence and the possible release of toxic by-products [20]. New strategies have to be devised for their application in deep tissue infection that require reconstructive surgeries.

In recent decades, 3D bioprinting has defined a new paradigm in regenerative medicine and tissue engineering approaches. A plethora of hydrogels (biomaterial inks) have been developed, which potentially can be laden with cells (then called “bioinks” when cells are included) and processed by 3D bioprinters to generate tissue substitutes of defined shape and structure, intended to be used clinically. In regard to skin tissue engineering, Cubo et al. have shown that fibrin based bioinks containing either fibroblasts or keratinocytes could be 3D bioprinted as bilayered structures to produce functional skin equivalents that can be potentially used in burn wound treatment [22]. When such bioprinted skin equivalents are implanted, the immediate challenge would be to avoid potential infections. Thus, the integration of antimicrobial delivery systems in such approaches would be imperative.

In the current work, we aimed to develop an antibiotic loaded hydrogel delivery system, generated by 3D core/shell extrusion printing that can be flexibly used to produce wound coverage or to be integrated in a tissue substitute. The 3D core/shell printing method combines two hydrogels by coaxial extrusion such that one hydrogel is encapsulated by the other, resulting in scaffolds with core/shell architecture of the strands [23]. The core hydrogel is loaded with antibiotics acting as a depot and the shell hydrogel provides mechanical stability and functions as a diffusion barrier. We hypothesize that by the choice of hydrogels of core and shell parts and the variation of thickness of the shell part, the release kinetics of loaded antibiotics can be modulated. As the base material, alginate (ALG) was used due to its excellent biocompatibility and easy processability. Because of its low immunogenic profile, alginate is routinely used to encapsulate human cells for tissue engineering applications [24,25]. Furthermore, it is approved for clinical use and many commercial formulations are already used in clinics as wound dressings and drug delivery systems [26,27]. For the shell, the alginate was blended with methylcellulose (MC) to achieve excellent printability by (temporary) enhancing the polymer content and viscosity; the alginate-methylcellulose (ALG-MC) blend was previously developed in our lab and its cell compatibility was proven for various cell types [28,29,30,31]. Additionally, several modifications of this blend for ex. with Laponite, human blood plasma and bioglass has shown its versatility and applicability for various 3D bioprinting applications [32,33,34]. Furthermore, methylcellulose is also used to treat dry eyes and as a thickener, emulsifier, and stabilizer for various pharmaceuticals [35]. As a third component, Laponite (LAP) should be introduced in the system, either as antibiotic loaded core or in combination with alginate and methylcellulose as shell. Laponite, a synthetic magnesium silicate nanoclay, was specifically chosen in this study due to its intrinsic high adsorptive properties, attributed to its disc shaped structure with positively charged rim and negatively charged plane [36]. In addition, due to the electrostatic self-assembly of Laponite particles, suspensions show typical characteristics of shear-thinning gels, making it suitable for extrusion printing [32]. Currently, due to the versatile properties of Laponite, it is extensively studied and developed as a biomaterial for drug delivery and bone tissue engineering applications [37,38]. It is hypothesized that inclusion of Laponite can sustain the release of loaded antibiotics. These biomaterial inks, i.e., alginate-methylcellulose (ALG-MC) and alginate-methylcellulose-Laponite (ALG-MC-LAP) were previously used in bioprinting including cells, hence they are also referred to as bioinks [28,30,31,32,33].

Antibiotics commonly used for treating soft tissue infections (vancomycin, clindamycin, and gentamicin) are loaded in the scaffolds during fabrication: Efficient antibiotic loading strategies in the core depot were initially developed and their suitability with core/shell 3D printing was evaluated. The release of loaded antibiotics through the shell was studied, whereby the effect of Laponite (when included in the shell) and thickness of the shell was explored.

## 2. Materials and Methods

### 2.1. Preparation of Shell Biomaterial Inks

Shell biomaterial inks were composed of alginate, methylcellulose and/or Laponite. Two separate stock solutions of 3 and 6% (*w*/*v*) of sodium alginate (from brown algae, 71238, Sigma-Aldrich, Steinheim, Germany) were prepared by dissolving autoclaved powder in sterile double distilled water (ddH_2_O). Biomaterial ink composed of alginate and methylcellulose (ALG-MC) was prepared as described previously [28]. Briefly, autoclaved methylcellulose powder (M0512, Viscosity: 4000 cP, Sigma-Aldrich, Steinheim, Germany) was added to a 3% alginate solution such that the final concentration of methylcellulose was 9% (*w*/*w*). Similarly, biomaterial ink composed of alginate, methylcellulose and Laponite (ALG-MC-LAP) was prepared by first making a blend of 3% alginate and 3% Laponite (Laponite XLG, BYK additives & instruments, Widnes, UK; autoclaved Laponite powder was added to 3% alginate solution and was stirred overnight); followed by addition of 6% methylcellulose. The prepared shell biomaterial inks were stored overnight at 4 °C to allow swelling of methylcellulose, and then used for fabrication of scaffolds by 3D core/shell printing (Section 2.4).

### 2.2. Preparation of Antibiotics Loaded Core Biomaterial Inks

Respective antibiotic powders (Vancomycin HCl, Clindamycin HCl and Gentamicin sulphate, all provided by Heraeus Medical, Germany) were first dissolved in ddH_2_O at a concentration of 20 mg/mL. Additionally, 6% (*w*/*v*) alginate sol in ddH_2_O was prepared. Antibiotic solutions were added drop wise to the stirring 6% alginate solution. An equal volume of both the solutions was used, so that the final concentration of the antibiotics and alginate in the resultant suspension was 10 mg/mL and 3% (*w*/*v*), respectively. The antibiotic loaded alginate solution was used as a core part to fabricate 3D core/shell scaffolds (Section 2.4).

### 2.3. Rheological Characterization of the Biomaterial Inks

The biomaterial inks were subject to dynamic and constant shear rate using a parallel-plate rheometer (Rheotest RN 4.1; RHEOTEST Medingen, Ottendorf-Okrilla, Germany). Antibiotic loaded alginate solution (500 µL), ALG-MC or ALG-MC-LAP (1 g) were placed in between the plates with a distance of 0.1 mm. To assess the shear thinning behaviour of the biomaterial inks, viscosity was measured, while shear rate at an increment of 1 s^−1^ was applied for 100 s. Similarly, to assess the effect of the addition of antibiotics to alginate and Laponite to ALG-MC, their viscosity was measured when a constant shear rate of 10 s^−1^ was applied for 100 s.

### 2.4. Fabrication of Scaffolds by 3D Core/Shell (C/S) Printing

The biomaterial inks were loaded into 10 mL plotting cartridges (Nordson, Westlake, OH, USA) which were fixed onto a core/shell adapter, attached to the print head of the BioScaffolder 3.1 (GeSiM mbH, Radeberg, Germany). The BioScaffolder was placed inside a laminar air flow cell culture bench, so that the printing process was performed under sterile conditions. Self-made coaxial needles were used; the inner cylindrical metal needle with an outlet diameter of 200 µm (Globaco, Roedermark, Germany) was connected to the cartridge filled with antibiotic-alginate suspension. The outer conical plastic needle with various outlet diameters (610 µm, 840 µm or 1200 µm; Globaco, Roedermark, Germany) was connected to the cartridge filled with ALG-MC or ALG-MC-LAP biomaterial ink. Both the biomaterial inks were simultaneously extruded such that ALG-MC or ALG-MC-LAP biomaterial ink (shell) encapsulates antibiotic-alginate suspension (core). 3D scaffolds were fabricated by meandering deposition of the extruded core/shell strand in a layer-by-layer fashion (0°/90° lay-down pattern, 4 parallel strand sections per layer) leading to a continuous single strand scaffold of 4 layers. After completion of the extrusion process, C/S scaffolds were immediately stabilized by alginate crosslinking in sterile 100 mM calcium chloride (CaCl_2_;Carl Roth, Karlsruhe, Germany) solution for 10 min, followed by washing with sterile 0.9% sodium chloride (NaCl; Sigma-Aldrich, Steinheim, Germany) solution to remove excess CaCl_2_.

### 2.5. Release of Antibiotics

Antibiotic-loaded C/S scaffolds with a size of approximately 14 × 14 × 3 mm (l × b × h) were put in 12 well plates containing 1 mL of 0.9% NaCl solution, so that the loaded antibiotics could be released; C/S scaffolds without antibiotic loading served as control samples. After time intervals of 2 and 6 h, 1, 2, 3, 7 and 14 d, the release solution was collected and fresh NaCl solution was added to the scaffolds. The released antibiotic solution was stored in sterile 1.5 mL tubes (Sarstedt AG, Nümbrecht, Germany) at 4 °C until used for quantification.

### 2.6. Spectrophotometric Quantification of Antibiotics

Vancomycin and clindamycin in solution were quantified by measuring their absorption at 230 and 198 nm, respectively, after determining their peak absorbance at these wavelengths. Pure vancomycin (0–250 µg/mL) and release solutions were loaded in a quartz 96-well microplate (Hellma Analytics, Müllheim, Germany) and the absorbance at 230 nm was measured using a spectrophotometer (Infinite^®^ M200 Pro, Tecan, Menendorf, Switzerland). Absorbance of pure clindamycin and release solutions was measured in quartz cuvettes (Hellma Analytics, Müllheim, Germany) at 198 nm using a spectrophotometer (Genesys 10 Bio UV-vis spectrophotometer, Thermo Fisher Scientific, Waltham, MA, USA). Gentamicin was quantified by using Ninhydrin assay [39]. Ninhydrin reagent (Sigma Aldrich, Steinheim, Germany) was added to pure gentamicin and release solutions and the reaction was performed according to manufacturer’s instructions. Absorbance of the reaction complex was measured at 570 nm using a spectrophotometer (Infinite^®^ M200 Pro, Tecan, Menendorf, Switzerland).

### 2.7. Agar Diffusion Assays to Quantify Antibacterial Activity of the Released Antibiotics

Agar diffusion assay (according to DIN 58940-3:2007-10) was adapted to allow quantifying the amount of antibiotics in the release solution. For preparation of the agar plates, Mueller-Hinton broth (21 g/L) was added to dissolved agar (15 g/L; both from Carl Roth, Karlsruhe, Germany) in equal volumes. The pH of the solution was adjusted to 7.2–7.4 by adding HCl or NaOH. After autoclaving (20 min at 121 °C) and cooling to approx. 70 °C, 20 mL of liquid Mueller-Hinton agar each time was dispensed into sterile Petri dishes (size: 92 × 16 mm; Sarstedt AG, Nümbrecht, Germany). Approximately 3 mm thick solidified agar was formed in the Petri dishes after cooling down to room temperature. The agar plates were stored in sterile plastic bags at 4 °C until further use. Bacterial suspensions of *S. aureus* (ATCC 27853) and *S. epidermidis* (ATCC 12228) with an OD600 of 0.1 corresponding to 107 CFU/mL were prepared in 0.9% NaCl solution. 2 mL of the bacteria suspensions were pipetted onto the agar surface and spread by moving the plates in circular motion for 30 s. The plates were then rested for 5 min to let the bacteria sediment onto the surface. Residual liquid was removed, and the plates were dried at room temperature for 10 min. Each plate was previously marked into 6 equal sectors to accommodate 6 samples (standard antibiotic concentrations or release solutions). Autoclaved Rotilabo^®^-sample carrier (Ø = 6 mm, thickness = 0.75 mm; Carl Roth, Karlsruhe, Germany) were placed at the approximate centre of each sector. 20 µL of defined antibiotic solutions or release solutions were pipetted on to the middle of the sample carrier. The plates were then incubated for 18 h at 36 °C so that the loaded antibiotics in the sample carrier could diffuse radially outwards. The plates were then imaged using a camera system (P.CAM360, SmartLab systems, TU Dresden, Dresden, Germany). A clear circular zone around the sample carrier is defined as the zone of inhibition (ZOI). Area of the ZOI is determined from the acquired images using ImageJ (1.52 h, National Institutes of Health, Bethesda, MD, USA). By comparing the ZOI areas determined for the release solutions with a standard curve obtained by plotting ZOI areas resulting from solutions with known antibiotic concentrations, the calculated effective concentration (CEC) of antibiotics in the release solutions was determined. To avoid deviations caused by batch variations of bacterial suspensions, Mueller-Hinton agar, etc., standard curves were determined for each set of experiments.

### 2.8. Statistical Analysis

All experiments were performed using replicates (*n* = 3–6, indicated in the figure captions). Results are presented as mean value ± standard deviation. One-way analysis of variance was used to evaluate statistical significance at a level of *p* < 0.05 (OriginPro 9.1, OriginLab, Northampton, MA, USA).

## 3. Results

### 3.1. Fabrication of Antibiotic-Loaded Scaffolds by Core/Shell Extrusion Printing

#### 3.1.1. Antibiotic-Loaded Core Biomaterial Inks

**Figure 1:**
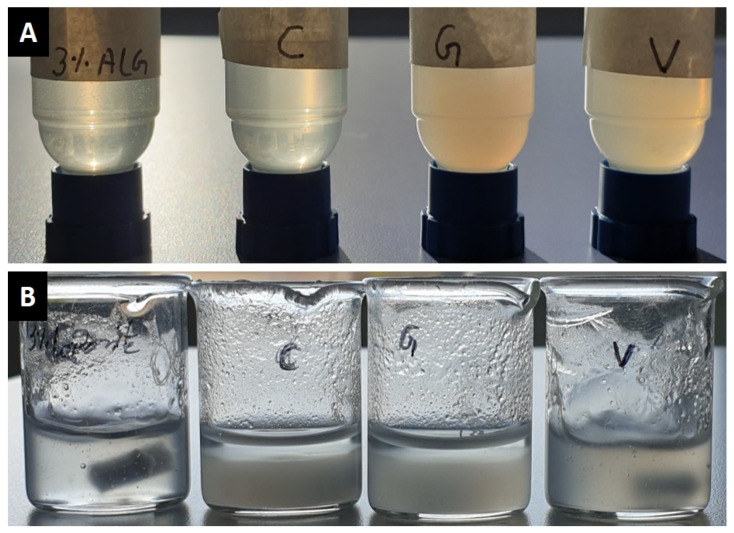
(**A**) Images of pure 3% alginate and of clindamycin, gentamicin, and vancomycin in 3% alginate at a concentration of 10 mg/mL (positioned left to right, respectively). (**B**) Images of pure 3% Laponite and of clindamycin, gentamicin, and vancomycin in 3% Laponite at a concentration of 10 mg/mL (positioned left to right, respectively). After adding 20 mg/mL antibiotic solution to 6% Laponite and stirring, a clear phase separation (after a few minutes at rest) of clindamycin and gentamicin was observed.

Attempts to directly dissolve the antibiotic powders in a 3% alginate solution resulted in the formation of agglomerates leading to clogging the 200 µm core needle during 3D printing. In an alternative approach, the antibiotics were first dissolved in ddH_2_O (two times concentrated) and then, equal amounts of these antibiotic solutions and 6% alginate solution were mixed while stirring. The resultant antibiotic-alginate suspensions did not form any agglomerates and had uniform consistency (Figure 1A), and hence were demonstrated to be suitable for 3D C/S printing. Antibiotics were also attempted to dissolve in low concentrated (1–3%) Laponite solutions, however, strong agglomerate formation as well as—in case of clindamycin and gentamicin—strong phase separation was observed (Figure 1B).

#### 3.1.2. Rheological Characterization of Core and Shell Biomaterial Inks

**Figure 2:**
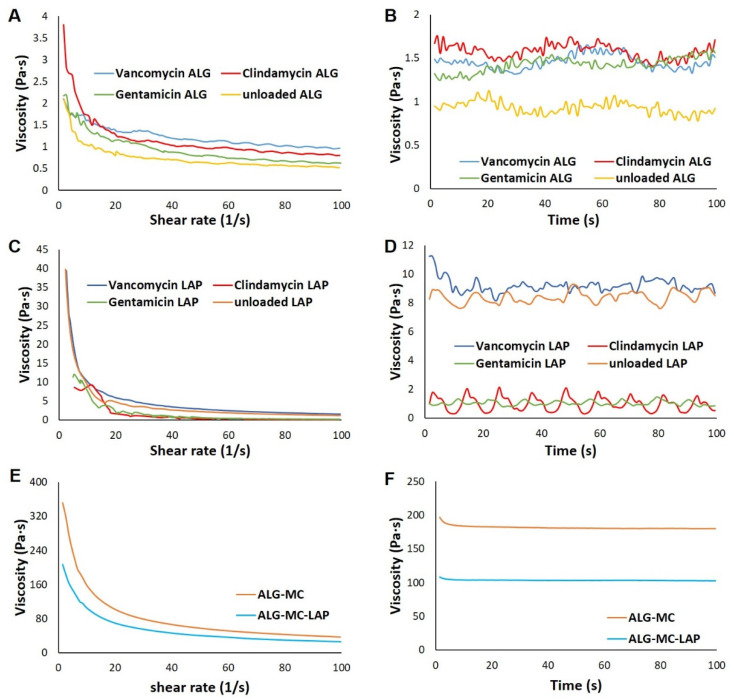
Rheological characterization of core and shell biomaterial inks. Representative plots showing the shear thinning behaviour of core—3% alginate and 3% Laponite loaded with antibiotics (**A**,**C**) and of shell—ALG-MC and ALG-MC-LAP (**E**). Viscosity of core and shell biomaterial inks, measured at a constant shear rate of 10 s^−1^ over a period of 100 s (**B**,**D**,**F**), respectively.

A significant difference in the viscosity of core and shell biomaterial inks was observed, however, all of them showed a shear thinning behaviour (Figure 2A,C,E). In case of the core biomaterial inks, a higher viscosity, though not significant, was observed for antibiotic loaded alginate when compared to unloaded alginate (Figure 2B), indicating that addition of antibiotics influenced the rheological properties of 3% alginate solution. Antibiotic loaded alginate showed shear thinning behaviour with clindamycin-alginate showing highest decrease in viscosity with increasing shear rate (3.24 ± 0.91 Pa·s; *n* = 3), followed by gentamicin-alginate (1.66 ± 0.08 Pa·s; *n* = 3), unloaded alginate (1.401 ± 0.14 Pa·s; *n* = 3) and vancomycin-alginate (1.26 ± 0.10 Pa·s; *n* = 3) (Figure 2A).

In case of antibiotic loaded Laponite, shear thinning behaviour was also observed, with vancomycin-Laponite showing highest decrease in viscosity with increasing shear rate (40.42 ± 2.61 Pa·s; *n* = 3), followed by unloaded Laponite (35.08 ± 3.21 Pa·s; *n* = 3), gentamicin-Laponite (11.98 ± 1.13; *n* = 3) and clindamycin-Laponite (6.76 ± 1.62 Pa·s; *n* = 3) (Figure 2C). Lower viscosity of clindamycin and gentamicin loaded Laponite (1.22 ± 0.30 Pa·s and 1.67 ± 0.45 Pa·s, respectively; *n* = 3) compared to vancomycin and unloaded Laponite (8.50 ± 0.59 Pa·s and 7.76 ± 0.60 Pa·s; respectively; *n* = 3) was observed (Figure 2D), indicating that clindamycin and gentamicin strongly interacted with Laponite. A strong phase separation and drastic reduction in viscosity of clindamycin and gentamicin loaded Laponite, along with agglomerate formation in vancomycin-Laponite, rendered antibiotic-Laponite biomaterial inks non-printable. Thus, these biomaterial inks were excluded from further investigations. The shell biomaterial inks ALG-MC and ALG-MC-LAP had shown shear thinning behaviour (Figure 2E) similar to those observed in our previous works [30,32]. The decrease in viscosity, during shear thinning measurements, of ALG-MC (282.5 ± 36.4 Pa·s; *n* = 3) was significantly higher than that of ALG-MC-LAP (191.7 ± 33.9 Pa·s; *n* = 3). Though total polymer/clay content of ALG-MC and ALG-MC-LAP was 12% (*w*/*v*), viscosity (measured at constant shear rate of 10 s^−1^; Figure 2F) of ALG-MC was significantly higher than those of ALG-MC-LAP (182.5 ± 16.4 Pa·s & 101.79 ± 2.09 Pa·s, respectively; *n* = 3).

#### 3.1.3. Extrusion-Printing of Antibiotic-Loaded Core/Shell Scaffolds

**Figure 3:**
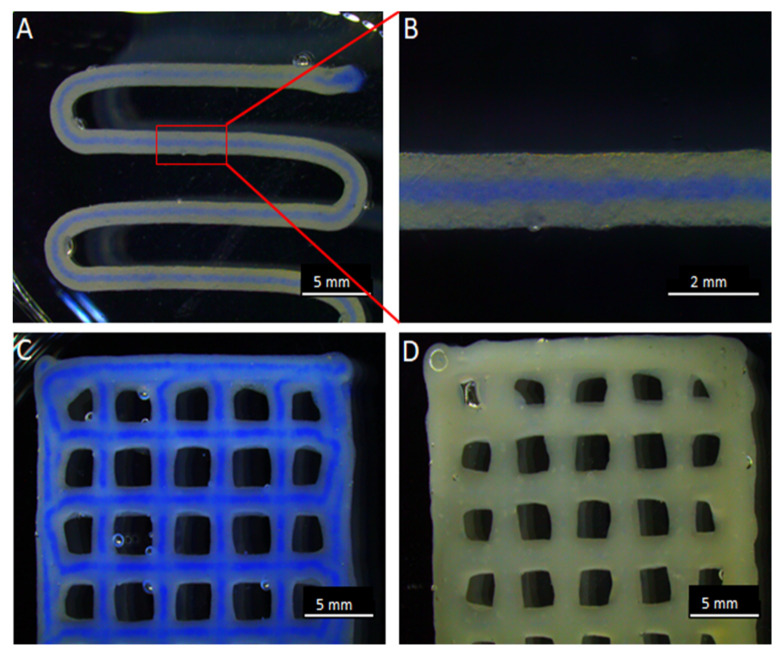
3D Core/shell printing. A C/S scaffold with the shell composed of ALG-MC-LAP and the core of 3% alginate, coloured with blue ink for visibility, after 1 layer of 3D printing (**A**). A magnified image of the C/S strand (**B**). C/S scaffold of 4 layers after finishing the 3D printing (**C**). A full strand (i.e., no core) 3D bioprinted scaffold of 4 layers (**D**).

As there is a significant difference in the viscosities of antibiotic loaded core and shell biomaterial inks, an ideal combination of extrusion pressure was needed to achieve similar flow rate of both core and shell biomaterial inks during 3D C/S printing. An extrusion pressure of 20–40 kPa for antibiotic-alginate suspension (core), 160–180 kPa for ALG-MC and 120–140 kPa for ALG-MC-LAP (shell biomaterial inks) was found to be optimal at an extrusion/printing speed of 5 mm/s. At these parameters, the core could be uniformly localised at the centre of the strand without rupturing the shell (Figure 3). After crosslinking alginate with CaCl_2_, the C/S scaffolds maintained their architecture for 14 d in 0.9% NaCl, independent of loaded antibiotics and shell material composition.

### 3.2. Spectrophotometric Methods for Quantifying Antibiotics in Release Solutions

**Figure 4:**
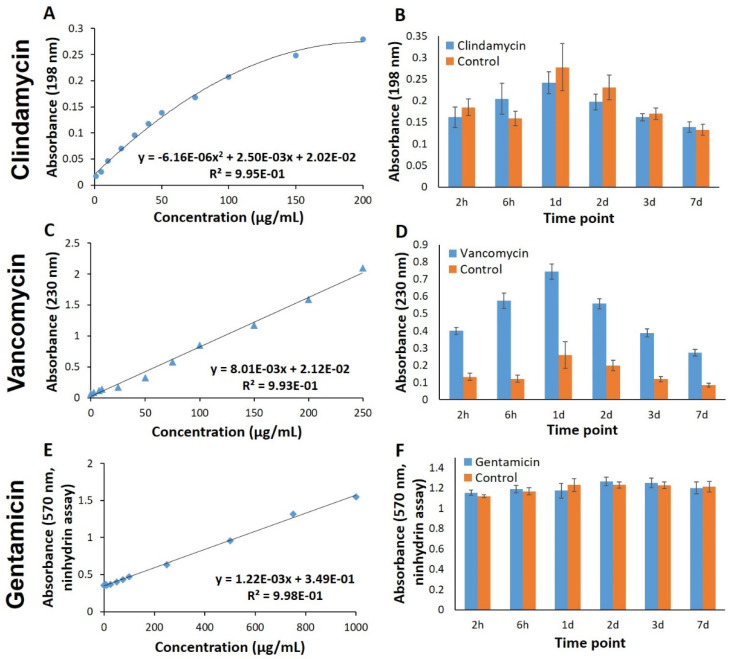
Spectrophotometric quantification of antibiotics. Standard curves (**A**,**C**,**E**) obtained by measuring the absorbance at 198 nm, 230 nm and Ninhydrin assay of defined concentrations of clindamycin, vancomycin and gentamicin in 0.9% NaCl solution, respectively. Analysis of the release solutions (**B**,**D**,**F**) obtained from antibiotic loaded and unloaded (control) ALG-MC scaffolds, using the respective method (*n* = 5).

Previously established methods, described in the literature, were initially used to quantify antibiotic concentrations in release solutions obtained from the ALG-MC scaffolds. With these spectrophotometric methods, measured absorbance of pure antibiotic solutions at specific excitation wavelengths resulted in very good fitted standard curves (Figure 4A,C,E). However, analyses of release solutions revealed considerable background signals caused by the presence of scaffold materials (Figure 4B,D,F). In case of vancomycin, a significantly higher absorbance (at 230 nm) of release solutions obtained from loaded vs. unloaded (“control”) ALG-MC scaffolds was observed (Figure 4D), thus, quantification of released vancomycin was possible to a limited extent. In contrast, in case of clindamycin quantification, absorbance measured at 190 nm of release solutions was higher for unloaded ALG-MC scaffolds when compared to clindamycin loaded scaffolds (Figure 4B). Additionally, in the Ninhydrin assay used to quantify gentamicin, a strong absorbance of release solutions obtained from unloaded ALG-MC scaffolds was found, which does not allow for the quantification of released gentamicin (Figure 4F). Thus, these methods were not suitable to investigate antibiotics release from the C/S scaffolds.

### 3.3. Agar Diffusion Assay for Antibiotics Quantification

**Figure 5:**
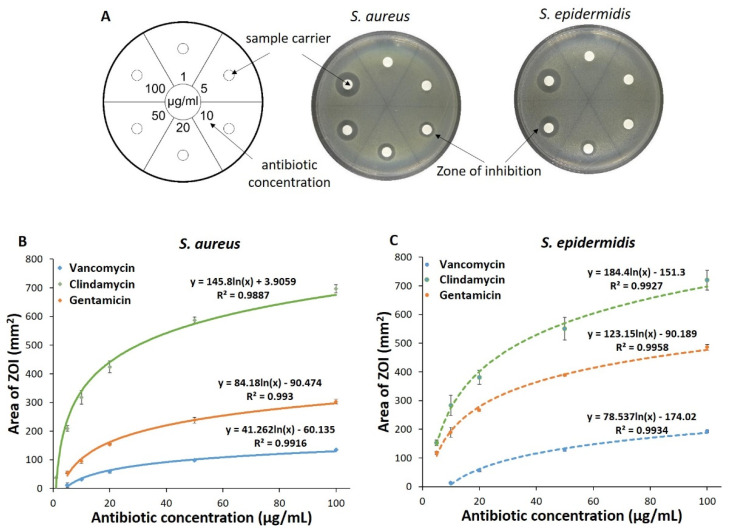
Adaptation of the agar diffusion test for quantification of antibiotics. A schematic diagram of an agar plate with sample carriers: Numbers in each sector indicate the concentration of antibiotic solution. Example images of the agar plates whose sample carriers were loaded with vancomycin, which led to formation of a clear area (ZOI) around the sample carriers (**A**). Standard curves of clindamycin, gentamicin and vancomycin obtained by plotting the area of ZOI developed on agar plates containing *S. aureus* (**B**) and *S. epidermidis* (**C**) vs. respective antibiotic concentration loaded on the sample carriers (*n* = 3).

As an alternative method, quantification of the effective concentration of antibiotics in the release solutions via its biological effect was evaluated. First, agar diffusion assays were carried out with the pure antibiotics dissolved in 0.9% NaCl at defined concentrations. Agar plates that had two bacterial species (*S. aureus* or *S. epidermidis*) developed clear areas, i.e., zones of inhibition (ZOI), around the sample carriers indicating that the loaded antibiotics had diffused radially outwards from the sample carriers and had killed the bacteria (exemplarily shown for vancomycin in Figure 5A). After quantifying the area of ZOI, it was found to be directly proportional to the concentration of loaded antibiotics. A plot of area of ZOI vs. effective antibiotic concentrations yielded a standard curve unique for the type of antibiotic and also for the type of bacteria (Figure 5B,C).

### 3.4. Release Kinetics of Antibiotics from C/S Scaffolds

**Figure 6:**
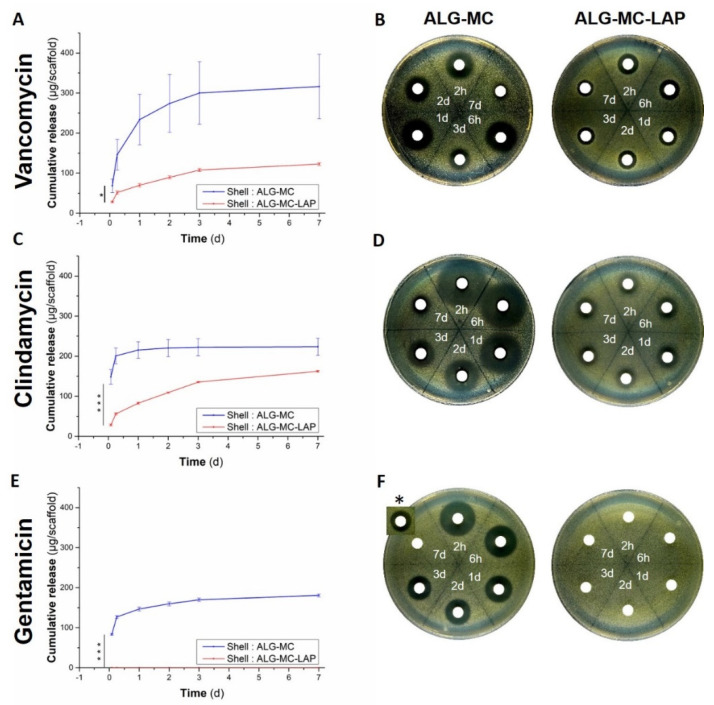
CEC of antibiotics from C/S scaffolds; impact of shell biomaterial ink composition. Cumulative CEC release of antibiotics loaded in ALG-MC and ALG-MC-LAP C/S scaffolds (printed with 200/840 C/S needles), quantified by agar diffusion assay (using *S. aureus* strain; **A**,**C**,**E**) over a period of 7 d. Statistical analysis performed only on burst release, i.e., release of antibiotics at 2 h (*n* = 3; * *p* < 0.05; *** *p* < 0.001). Representative images of ZOI formed on the agar plate when release solutions were added to sample carriers (**B**,**D**,**F**). A cropped image of ZOI of d 7 release sample of gentamicin was performed on a different agar plate and is added onto the ZOI of remaining samples, * in (**F**).

Effective concentration of the antibiotics released from the loaded scaffolds over time was quantified using the respective standard curves obtained in agar diffusion assay of both the bacterial species (*S. aureus* and *S. epidermidis*; Section 3.3). In general, release solutions obtained at all time points and scaffold variants (except for ALG-MC-LAP with gentamicin-alginate in the core) used in the study developed ZOI for both the bacterial species, indicating that the released antibiotics maintained their potency. It was observed that the ZOI area (developed for the same concentration of antibiotic or release solution) was higher for *S. aureus* as compared to *S. epidermidis* (Figure 6 and Figure S1, respectively).

#### 3.4.1. Impact of Shell Composition

Antibiotic loaded core biomaterial inks were combined with either ALG-MC or ALG-MC-LAP as shell; both types of scaffolds were fabricated using a 200/840 core/shell needle. The cumulative release of antibiotics (calculated effective concentration; CEC), as determined by using *S. aureus* and *S. epidermidis*, are depicted in Figure 6 and Figure S1, respectively; Table S1, Supplementary Materials shows the CEC of released antibiotics at the respective time points (absolute release), as determined by using *S. aureus*.

Release solutions collected from ALG-MC scaffolds at initial time points (2 h and 6 h) developed large ZOI, indicating a burst release of the loaded antibiotic. For release solutions collected at later time points, a progressive decrease in the developed ZOI was observed. Importantly, even though a higher burst release of antibiotics from ALG-MC scaffolds was observed, a ZOI still developed for release solution collected after 7 day, indicating that the released antibiotics maintained their potency at least till day 7 (Figure 6B,D,F). The release of loaded antibiotics from ALG-MC-LAP scaffolds was significantly lower and more evenly over the 7 day compared to ALG-MC scaffolds, indicating that Laponite in the shell arrests the antibiotics diffusion. In case of gentamicin loaded ALG-MC-LAP scaffolds, no release was observed, indicating a complete arrest of gentamicin diffusion in the shell.

Comparison of the different antibiotics quantified in the release solutions of ALG-MC scaffolds revealed that initial burst release (release solution collected after 2 h) of clindamycin (148.23 ± 18.34 µg/mL) was significantly higher compared to gentamicin (83.09 ± 2.05 µg/mL) and vancomycin (68.3 ± 17.1 µg/mL). At later time points, the average release (from d 2 till d 7) of clindamycin (2.7 ± 1.7 µg/mL) was significantly lower than gentamicin (11.31 ± 1.15 µg/mL) and vancomycin (27.4 ± 9.9 µg/mL). Quantification of vancomycin and clindamycin in release solutions obtained from ALG-MC-LAP scaffolds revealed a sustained release of both the antibiotics over the observation period of 7 day (average release per day for clindamycin and vancomycin was 27.03 ± 0.83 µg/mL and 20.44 ± 4.26 µg/mL, respectively).

The same release solutions that were used in agar diffusion assay with *S. aureus* strain were also used for *S. epidermidis*: A similar trend in CEC of vancomycin and gentamicin from release solutions obtained from both ALG-MC and ALG-MC-LAP scaffold types was observed with *S. epidermidis* (Figure S1). Notably, total clindamycin release from ALG-MC scaffold was significantly lower when quantified using *S. epidermidis* strain compared to *S. aureus* strain (67.61 ± 6.60 µg/mL and 223.32 ± 21.35 µg/mL, respectively) in the agar diffusion assay. Moreover, clindamycin release solutions obtained from ALG-MC-LAP scaffolds did not result in ZOI formation (Figure S1C,D).

#### 3.4.2. Impact of Shell Thickness

**Figure 7:**
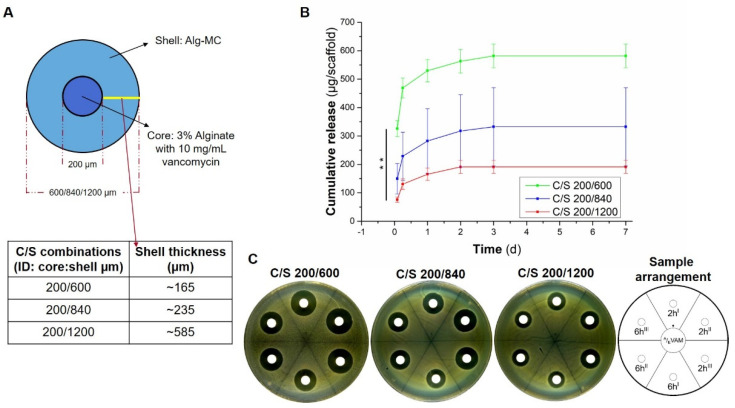
Release of vancomycin from C/S scaffolds: impact of shell thickness. Schematic diagram showing different coaxial needle combinations (**A**). Release of vancomycin from ALG-MC scaffolds having different shell thickness (*n* = 5; ** *p* < 0.005) (**B**). Statistical analysis performed only on burst release, i.e., release of antibiotics at 2 h (*n* = 3; ** *p* < 0.005). Representative images of ZOI formed in the presence of release solutions collected from scaffolds with different shell thickness after 2 h and 6 h, respectively (**C;** release solutions obtained from three scaffolds at respective time point were loaded on the sample carrier, denoted by I, II, III).

C/S scaffolds of ALG-MC (with vancomycin-alginate core) with different shell thickness were fabricated by using coaxial needles having a combination of inner needle with constant outlet diameter of 200 µm with outer needles with varying outlet diameters of 600 µm, 840 µm and 1200 µm. Thus, a theoretical shell thickness of ~165 µm, 235 µm and 585 µm, respectively, was achieved (Figure 7A). Initial burst release of vancomycin was found to be inversely proportional to the shell thickness of the scaffolds, i.e., 325.82 ± 27.91 µg/mL, 149.4 ± 58.2 µg/mL and 75.74 ± 9.25 µg/mL for needle combinations of 200/600, 200/840 and 200/1200 µm, respectively (Figure 7B,C). Additionally, the total amount of vancomycin released over a period of 7 d was highest for scaffolds fabricated by using a 200/600 µm combination coaxial needle (581.50 ± 41.59 µg/mL), followed by 200/840 µm (332.3 ± 137.7 µg/mL) and 200/1200 µm (191.01 ± 22.89 µg/mL).

## 4. Discussion

In the current work, alginate-based hydrogels, along with the 3D core/shell extrusion printing method, were used to develop a hydrogel based antibiotic delivery system intended for treating soft tissue infections in open fractures and burn wounds. The spatially separated organization of low viscous antibiotic loaded hydrogel within a high viscous hydrogel, i.e., in core/shell architecture, has three distinctive advantages: (1) The core compartment serves as a depot of antibiotics. Varied concentrations of antibiotics, according to the clinical demand, can be loaded into the core and the core material can be adapted to allow efficient loading with antibiotics, without the need of strongly considering shape fidelity after printing. The latter is ensured by the shell material that additionally acts as a diffusion barrier for antibiotics loaded in the core, leading to their retention and release over a longer time. Furthermore, by varying the shell thickness, the release of loaded antibiotics can also be tuned. (2) A variety of biomaterials can be flexibly combined to fabricate core/shell scaffolds, thus this fabrication method can be adapted to various target tissues, for example, also to fabricate scaffolds intended for bone tissue engineering. Additive effects of intrinsic advantages of both the biomaterials used in 3D core/shell printing can be expected. In our present study, changing the composition of the shell components, i.e., the addition of Laponite to ALG-MC to shell biomaterial ink resulted in the reduction of burst release and enhanced sustained release behaviour of the loaded antibiotics, implying that the intrinsic adsorptive property of Laponite played a deterministic role in tuning the release kinetics. Additionally, other scaffold properties can be tailored: In another example showing such a synergy, Raja et al. demonstrated that core/shell scaffolds composed of brittle ceramic (α-TCP) core and 6% alginate (softer) shell had significantly better mechanical properties compared to pure alginate scaffold and were less brittle compared to an exclusive ceramic scaffold [40]. In our ongoing work, we are developing a core/shell scaffold system composed of a soft hydrogel loaded with sensitive biological components as the core and self-setting calcium phosphate cements as the shell (Figure S2), intended to be used for bone tissue engineering applications. These C/S scaffolds, having clinically relevant sizes, could sustainably release loaded biological components and maintain their bioactivity in the long term and also had mechanical properties comparable to trabecular bone. (3) As 3D core/shell printing uses two biomaterials, differential release of multiple antibiotics and/or other biological components when loaded in core and/or shell can be achieved [23]. Additionally, stimuli responsive release systems can be developed as shown by Wei et al. [41] where photothermal effects of polydopamine (present in the shell) was employed to thermally reduce the viscosity of drug loaded gelatin upon irradiating the scaffold with near infrared laser, thus releasing the drug. Upon withdrawal of the laser, drug loaded gelatin retained its gel characteristics, thus arresting the release. Hence, core/shell scaffolds can be designed and fabricated according to the needs of the treatment required for a patient.

### 4.1. Loading of Antibiotics in Core Biomaterial Inks

To prepare antibiotic loaded core biomaterial ink, a simple approach of dissolving antibiotic powder in a 3% alginate solution was not feasible, especially for clindamycin and gentamicin, as they formed agglomerates. The alternative approach, where two times concentrated antibiotic solution was slowly added to a continuously stirred two times concentrated alginate solution, was successful: The addition of clindamycin to alginate yielded a clear solution, indicating that it had completely dissolved in alginate. A translucent solution of vancomycin and gentamicin alginate indicates that these antibiotics were not completely soluble in alginate. Heriot et al. observed the development of opaque alginate gel beads when incubated in gentamicin solutions [42]. This is also supported by Gordon et al.’s work, where precipitation was observed when alginate was in contact with gentamicin for at least 2 h [43]. All these observations, taken together, hint at weak electrostatic and/or intermolecular interactions between alginate and the investigated antibiotics, whose degree varied among the different antibiotics. However, the addition of all three antibiotics did not significantly alter the viscosity or the shear thinning behaviour of the biomaterial inks (Figure 2A,C), indicating that they do not have a crosslinking effect on alginate. Attempts to dissolve clindamycin and gentamicin in 3% Laponite resulted in strong phase separation (Figure 1B) and drastic reduction of viscosity of the resultant solution (Figure 2D), indicating that these two antibiotics strongly interacted with Laponite. Additionally, when vancomycin was added to Laponite, agglomerate formation was observed. Therefore, 3D C/S printing was not possible with these mixtures. The drastic reduction in viscosity when clindamycin and gentamicin were added to Laponite could be attributed to the disruption of the “house of card” assembly of Laponite nano-discs; probably due to strong electrostatic attraction between the face of the nanodiscs and clindamycin or gentamicin molecules [44]. Additionally, the phase separation observed for clindamycin- and gentamicin-Laponite could be attributed to the strong electrostatic interactions. In case of vancomycin-Laponite, agglomerate formation could be a result of strong electrostatic repulsion between the nano discs and vancomycin molecules. It can be speculated that, owing to steric hindrance of high molecular weight vancomycin molecules, the house of cards assembly of the nanodiscs might have been retained, hence vancomycin-Laponite had a similar viscosity and shear thinning behaviour (Figure 2C,D).

Both shell hydrogel blends, i.e., ALG-MC and ALG-MC-LAP, could be easily extruded through the coaxial needle with the thinnest shell thickness (i.e., ~165 µm; Figure 6A) used in this study. Additionally, both the shell biomaterial inks rendered high shape fidelity to C/S scaffolds, though the core was a low concentrated (3% alginate) hydrogel. Similar to our previous work [32], the shell biomaterial inks ALG-MC and ALG-MC-LAP showed strong shear thinning behaviour. However, the viscosity of ALG-MC/LAP was found to be lower than ALG-MC at a constant shear rate of 10 s^−1^; an opposite observation with respect to our previous work. This can be attributed to the different swelling regimes for MC (overnight at 4 °C in this study compared to 2 h at room temperature in our previous work), the main component that gives the biomaterial inks their high viscosity. However, both the biomaterial inks were found perfectly suitable for 3D printing and maintained high print fidelity. Additionally, high printing speed of 5 mm/s could be achieved, thus relatively large scaffolds (~14 × 14 × 3 mm; Figure 2) could be fabricated in less than 2 min.

### 4.2. Establishing Suitable Antibiotic Quantifying Methods

Spectroscopic quantification methods mentioned in the literature were initially used to quantify antibiotics in the release solutions. A reliable standard curve for pure antibiotics in saline could be developed (Figure 3). However, absorbance measurements of release solutions obtained from unloaded samples indicated the presence of high amounts of antibiotics; in case of clindamycin and gentamicin, the values were close to those of the antibiotic loaded samples. It was postulated that high absorbance obtained for unloaded sample release solutions was caused by scaffold components, i.e., alginate and methylcellulose. This observation was confirmed by measuring the absorbance of pure alginate and methylcellulose solutions (data not shown). MC is an inverse thermal gelling biopolymer, i.e., it is in gel form at higher temperatures and soluble at lower temperatures. The gelation temperature depends on the MC concentration, molecular weight, and the electrolyte concentration [29]. For ALG-MC scaffolds, which were incubated—as in the present study—in CaCl_2_ solution for alginate crosslinking, we have shown that methylcellulose is partially released [29]. Thus, it can be postulated that at least the release of methylcellulose from the scaffolds might have resulted in strong absorbance at 198 nm and 230 nm. Consequently, spectroscopic antibiotic quantification methods were unsuitable for the current study due to its rather unspecific measuring principle. Alternatively, more specific methods such as immunoassay liquid chromatographic and mass spectrometric methods for quantification of antibiotics in complex solutions [45] could be employed instead. However, owing to extensive sample preparation and the high technical effort required for these methods and the very high number of samples obtained in our experiments, these methods were considered impractical for the current study.

Microbiological assay, a classic method, has been traditionally used to quantify MICs of various antibiotics [46]. The quantification of antibiotics is based on the response of bacteria used in these assays. The area of ZOI is directly proportional to the amount of antibiotic in the sample, and no specific response from scaffold components was seen. This assay could semi-quantitatively provide information about the concentration of antibiotics in complex samples [47,48]. In the current study, agar diffusion assay was employed to quantify the released antibiotic from the scaffolds as (1) interference from scaffold components was not observed, (2) semi-quantitative determination of released antibiotics could be performed and (3) simultaneously, potency of the released antibiotics could be tested on bacterial species usually found in burn wounds (i.e., *S. aureus* and *S. epidermidis*). An increase in area of ZOI was observed when increasing antibiotic concentrations were loaded onto the sample carrier (Figure 4). Additionally, a standard curve could be generated by plotting the area of ZOI vs. concentration of antibiotic loaded onto the sample carrier (Figure 4C,D). Once the standard curve was developed, an area of the ZOI’s formed for release solutions was used to determine the antibiotic concentration.

### 4.3. Release of Antibiotics from Core/Shell Scaffolds

During the release studies performed for seven days, it was observed that all types of C/S antibiotic loaded scaffolds were stable and maintained their structure, proving that the dense shell provided structural stability to the scaffolds. Effective concentration of released antibiotics from the scaffolds over a period of seven days was quantified by agar diffusion assays. A disadvantage of employing this method is that absolute antibiotic concentration in the release solutions could not be quantified, on the other hand, potency (and effective concentration) of the released antibiotics was directly confirmed in this assay. It can be postulated that the diffusion of antibiotics majorly determined their release kinetics from ALG-MC scaffolds, hence the intrinsic molecular properties of the antibiotics and scaffold porosity must play a dominant role in determining the release kinetics of the loaded antibiotics. Additionally, molecular interactions between loaded antibiotics and scaffold components (i.e., alginate, methylcellulose and Laponite) must have an influence on their release kinetics. For ALG-MC scaffolds loaded with antibiotics, the average burst release (at 2 h) of clindamycin was significantly higher compared to vancomycin and gentamicin (Figure 6). Comparison of the molecular weight of the loaded antibiotics (Table 1) suggests that it plays a dominant role in the early phase of their release. Especially, the high molecular weight of vancomycin must have hindered their diffusion from the ALG-MC shell. The decrease in burst release of vancomycin observed with increasing shell thickness (Figure 7) further strengthens this hypothesis. On the other hand, clindamycin and gentamicin have similar molecular weights (Table 1); therefore, the difference in release kinetics, i.e., the higher initial burst release, as well as the higher total amount of release observed for clindamycin vs. gentamicin, must be attributed to other reasons: A strong interaction between gentamicin and alginate [42,49], resulting in an electrostatic attraction, might have hindered its release, whereas, in case of clindamycin, electrostatic repulsion between high amount of negatively charged hydroxyl groups and negatively charged alginate probably resulted in a very high burst and total release. In a study performed to evaluate the effect of vancomycin released from various viscous media on *S. aureus*, Kostenko et al. postulated that aromatic amino residues could have interacted by means of hydrogen bonds, with the carboxylic groups of alginates, whereas methylcellulose remained non interactive with vancomycin [50]. To the best of our knowledge, no studies have shown direct interaction of clindamycin and gentamicin with methylcellulose. Hence, at least for vancomycin, no interaction with methylcellulose can be expected. In general, the release kinetics of antibiotics loaded in ALG-MC scaffolds can thus be speculated to be highly dependent on molecular weight and interactions of antibiotics with alginate along with the shell porosity.

In contrast to the high variability in the amount of released antibiotics at different time points from ALG-MC scaffolds, consistent amounts of antibiotics were released from ALG-MC-LAP scaffolds over time. Thus, electrostatic interactions between Laponite and antibiotic molecules must have played a major role in their release. Addition of Laponite lowered the release of vancomycin and clindamycin and in the case of gentamicin, a complete arrest was observed. This observation can be attributed to the high adsorptive property of Laponite particles, consisting of a layered silicate structure housing a central magnesium core (disc shaped, with a size of about 25 nm in diameter and 1 nm in thickness), which has a highly negatively charged face and a positively charged rim [52]. High pKa values of gentamicin (Table 1; pKa: strongest Acidic, Strongest Basic = 12.55, 10.18, respectively) implies that it exists in a protonated state having a net positive charge, which is strongly attached to the negative surface of Laponite. Thus hindering, (probably completely arresting) gentamicin release from ALG-MC-LAP shell. Thus, total net charge of the antibiotics (as a result of their pKa or pI values) must have a dominant role in determining their release kinetics from ALG-MC-LAP. However, concentration of Laponite in the shell can be specifically adapted to be able to achieve desired controlled and/or sustained release of loaded antibiotics. Previous work of Hodder et al. showed release of methylcellulose (~25%) and calcium ions (therefore probably also release of alginate) from alginate-methylcellulose scaffolds over a period of 21 days [29]. Based on these findings, it can be speculated that remaining antibiotics (probably also gentamicin) and Laponite could be released at a later time.

Delivery of antibiotics from hydrogels (such as alginate, gelatin, chitosan, modified hyaluronic acid, etc.) have been extensively studied and their applicability for treating infections in open fractures and burn wounds was evaluated [19,53,54,55]. However, in most of these studies, antibiotics were loaded in either beads, membranes, or thick hydrogels. Various antibiotics are loaded by direct dissolving in hydrogels [19,53,56], absorbed [21,54,55,57,58] or as particles (preloaded in suitable carrier particles) [59] composed of hydrogels. Similar to our study, the release of the loaded antibiotics in these studies was also governed by their diffusivity, variable porosity of the hydrogel and their interaction with the hydrogel components. To the best of our knowledge, very few studies have been performed that focus on 3D printing of antibiotic loaded hydrogel scaffolds [60,61]. Kumari et al. developed 3D bioprinted scaffolds composed of recombinant spider silk protein incorporated with antibiotic loaded mesoporous silica nanoparticles. Release of antibiotics, studied in 2D films, from their hydrogel composite showed varied release kinetics depending on the release medium (for gentamicin ~34% and <5% in PBS and Tris/HCl, respectively), indicating that the release medium could have a drastic effect on antibiotics release. Additionally, the very low and sustained release of loaded antibiotics in PBS can be attributed to the diffusion barrier offered by the mesoporous silica nanoparticles [60]. Aldrich et al. fabricated a hybrid scaffold consisting of a hard polycaprolactone/ hydroxyapatite composite (PCL/HA) and a soft hydrogel (methacrylated hyaluronic acid and gelatin) using a two-channel 3D bioprinter. A burst release of daptomycin, loaded in the hydrogel, was observed, and reached a plateau at later points. The authors in this work have further demonstrated that bone marrow derived macrophage cells could be printed on the hydrogel strands of the scaffold, which was then used in mouse model study [61].

Similar to the study of Aldrich et al., the core/shell 3D printing method can be used to incorporate antibiotics or drugs in the core and viable cells in the shell compartment (or vice versa) to deliver both at the defect site. Additionally, the combination of antibiotics such as of gentamicin, vancomycin and clindamycin, proven for efficient antibacterial activity in bone related infections [62], can be loaded in different compartments of core/shell scaffolds to attain the desired release kinetics. Furthermore, the 3D C/S printing method can be combined with other biofabrication methods such as traditional 3D printing/bioprinting or inkjet bioprinting to develop hybrid scaffolds, to further aid in soft tissue regeneration. For example, methods of Cubo et al. [22], Kim et al. [63] and Turner et al. [64], where functional human skin grafts, muscle, and vascular constructs, respectively, are fabricated by 3D bioprinting, can be easily integrated with the current method; such that a skin or muscle substitute with antibiotics can be precisely manufactured and tailored to the needs of the patient. Alternatively, collagen gels (with or without cells) can be casted on core/shell antibiotic loaded scaffolds to develop a hybrid scaffold that can potentially aid in soft tissue regeneration and simultaneously avoid or treat infections.

## 5. Conclusions

Using 3D core/shell extrusion printing, a unique antibiotic delivery system has been developed, wherein a high viscous (shell) hydrogel encapsulates the low viscous antibiotic loaded (core) hydrogel. The shell component provides mechanical stability to the scaffolds and can modulate the release kinetics of the loaded antibiotic. Microbiological agar diffusion assay was adapted to quantify and simultaneously assess the potency of the released antibiotics. A sustained and potent release of the tested antibiotics (vancomycin, clindamycin, gentamicin) was observed for seven days. We demonstrated that by changing the composition of the shell hydrogel, i.e., by adding Laponite to ALG-MC, the release kinetics can be significantly slowed down. Additionally, by changing the shell thickness, the release of antibiotics can be modulated. Further work can focus on developing hybrid constructs, aiming at the integration of core/shell antibiotic loaded scaffolds with soft tissue regeneration. With many possibilities to tailor release kinetics of loaded antibiotics, such a versatile antibiotic delivery system could potentially open new treatment procedures for soft tissue infections and regeneration in open fractures and burn wounds.

## Figures and Tables

**Table 1:** Summary of the different properties of antibiotics used in this study. (NA = not available).

Properties	Vancomycin	Clindamycin	Gentamicin
Molecular weight (g/moL)	1485.7	461.4	575.67
Isoelectric point (pI)	8.1 [51]	NA	9.5 [51]
pKa (strongest acidic) *	2.99	12.16	12.55
pKa (strongest basic) *	9.93	7.55	10.18

* Predicted values; source: https://hmdb.ca/ (accessed on 2 August 2021).

## Data Availability

The data supporting this article are shown as figures in the results and Supplementary Materials. Raw datasets analysed in the present study are available from the corresponding author upon reasonable request.

## Supplementary Materials

The following are available online at https://www.mdpi.com/article/10.3390/pharmaceutics13122151/s1, Table S1: Absolute release of various antibiotics from ALG-MC and ALG-MC-LAP scaffolds quantified by agar diffusion assay (using *S. aureus*) during the release study, Figure S1: Release of antibiotics from C/S scaffolds; impact of shell biomaterial ink composition, and Figure S2: An image of a core/shell 3D printed calcium phosphate cement scaffold.
