# Supplementary material for: Controlled and Local Delivery of Antibiotics by 3D Core/Shell Printed Hydrogel Scaffolds to Treat Soft Tissue Infections
Source: Pharmaceutics. 2021 Dec 14;13(12):2151. doi: 10.3390/pharmaceutics13122151 (PMC8705560; doi:10.3390/pharmaceutics13122151)
Supplement: Supplementary file 1 [file pharmaceutics-13-02151-s001.zip › pharmaceutics-1451405-supplementary.pdf]

pharmaceutics

Supplementary Materials: Controlled and Local Delivery of Antibiotics by 3D Core/Shell Printed Hydrogel Scaffolds to Treat Soft Tissue Infections

Ashwini Rahul Akkineni, Janina Spangenberg, Michael Geissler, Saskia Reichelt, Hubert Buechner, Anja Lode and Michael Gelinsky

Table S1. Absolute release of various antibiotics from ALG-MC and ALG-MC-LAP scaffolds quantified by agar diffusion assay (using *S. aureus*) during the release study.

Antibiotic Type	Time Point	Shell: ALG-MC Average Amount of Antibiotics Released (mean ± standard deviation)	Shell: ALG-MC-LAP Average Amount of Antibiotics Released (mean ± standard deviation)
Vancomycin	2h	68.30 ± 17.05	28.30 ± 2.02
	6h	77.66 ± 21.18	23.16 ± 1.96
	1d	87.90 ± 28.60	18.36 ± 0.38
	2d	40.09 ± 11.64	19.63 ± 0.54
	3d	26.28 ± 6.03	18.26 ± 0.28
	7d	15.90 ± 2.71	14.93 ± 0.40
Clindamycin	2h	148.23 ± 18.34	28.42 ± 2.08
	6h	52.37 ± 7.77	27.48 ± 1.10
	1d	14.71 ± 1.12	26.82 ± 0.57
	2d	5.04 ± 0.44	26.28 ± 1.51
	3d	1.91 ± 0.11	26.17 ± 1.32
	7d	1.03 ± 0.02	26.86 ± 0.53
Gentamicin	2h	83.09 ± 2.05	0 ± 0
	6h	43.66 ± 2.04	0 ± 0
	1d	19.97 ± 1.31	0 ± 0
	2d	12.90 ± 0.48	0 ± 0
	3d	10.17 ± 0.70	0 ± 0
	7d	10.88 ± 1.10	0 ± 0

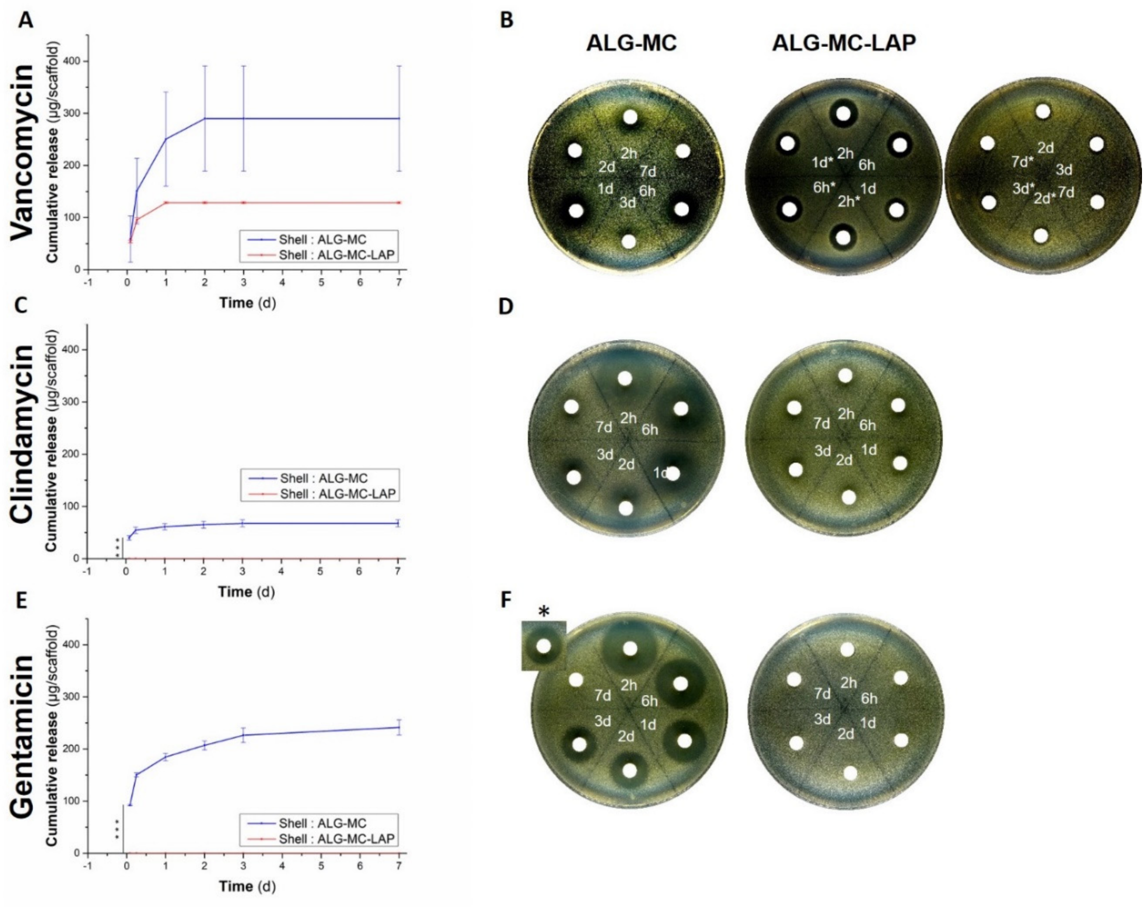

Figure S1. Release of antibiotics from C/S scaffolds; impact of shell biomaterial ink composition. Cumulative release of antibiotics loaded in ALG-MC and ALG-MC-LAP C/S scaffolds, quantified by agar diffusion assay (using S. *epidermidis* strain; **A, C & E**) over a period of 7 d (n=3), *** $p < 0.001$. Representative images of ZOI formed on the agar plate when release solutions were added to sample carriers (**B, D & F**). Two agar plates were used for release solutions obtained from vancomycin loaded ALG-MC/LAP scaffolds (**B**). A cropped image of ZOI of d 7 release sample of gentamicin was performed on a different agar plate and is added onto the ZOI of remaining samples (***** in **F**).

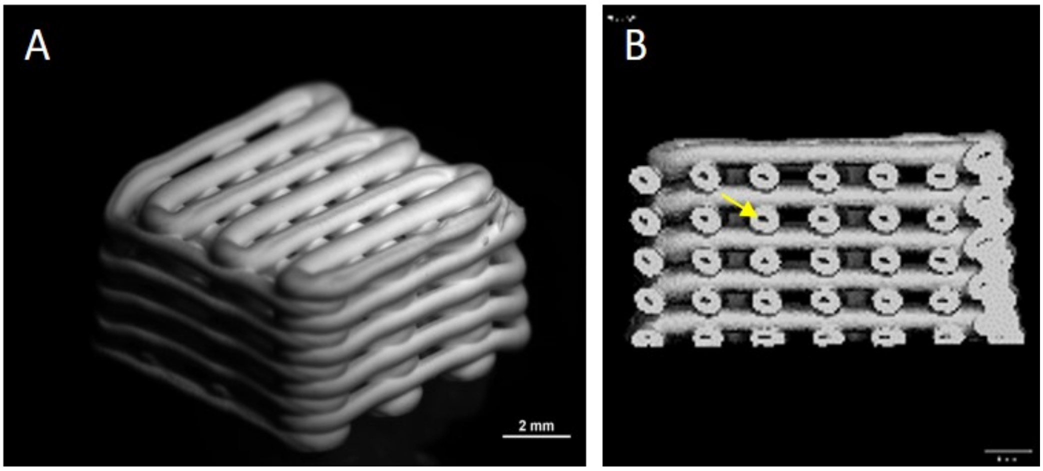

Figure S2. An image of a core/shell 3D printed calcium phosphate cement scaffold (**A**). Micro CT image of the scaffold showing the core/shell architecture of the strands (**B**); arrow indicating the hydrogel core of a strand. (visible as hollow lumen in µCT images).
